# Hepatocellular Early Apoptosis Associated with HES 130/0.4 Administration for Volume Replacement in Pigs After Severe Bleeding

**DOI:** 10.3390/vetsci12090787

**Published:** 2025-08-22

**Authors:** Helena Vala, Ana I. Faustino-Rocha, Rita Cruz, Carlos Venâncio, Aura Silva, João R. Mesquita, Ana Liza Ortiz, David A. Ferreira

**Affiliations:** 1 Research Center for Natural Resources, Environment and Society (CERNAS), Polytechnic Institute of Viseu, 3504-510 Viseu, Portugal; 2 Centre for the Research and Technology of Agro-Environmental and Biological Sciences (CITAB), Inov4Agro, 5000-801 Vila Real, Portugal; 3 University of Évora, Comprehensive Health Research Center (CHRC), 7004-516 Évora, Portugal; 4 University of Évora, School of Science and Technology, Department of Zootechnics, 7004-516 Évora, Portugal; 5 Epidemiology Research Unit (EPIUnit), Institute of Public Health, University of Porto, 4050-313 Porto, Portugal; 6 Agrarian School of Viseu (ESAV), Polytechnic Institute of Viseu, 3504-510 Viseu, Portugal; 7 Department of Zootechnics, University of Trás-os-Montes and Alto Douro, 5000-801 Vila Real, Portugal; 8 Portuguese Research Centre for Sustainable Chemistry (REQUIMTE), Faculty of Pharmacy, University of Porto, 4050-313 Porto, Portugal; 9 Abel Salazar Institute of Biomedical Sciences (ICBAS), University of Porto, 4050-313 Porto, Portugal; 10 Department of Veterinary Medicine, University of Cambridge, Cambridge CB2 ITN, UK; 11 University of Évora, School of Science and Technology, Department of Veterinary Medicine, 7004-516 Évora, Portugal

**Keywords:** apoptosis, hemorrhage, HES 130/0.4, histopathology, liver

## Abstract

Hydroxyethyl starch (HES) 130/0.4 is commonly used for volume replacement, yet its hepatic effects in the context of acute haemorrhage remain unclear. This study aimed to evaluate hepatic histopathological changes related to HES 130/0.4 administration when compared to Ringer’s lactate (RL) in healthy pigs subjected to acute bleeding under general anaesthesia. Eighteen pigs were randomised into three groups: RL (*n* = 6), HES 130/0.4 (*n* = 6), and a non-bleeding control (*n* = 6). Liver tissue was collected postmortem and analysed using haematoxylin–eosin staining, cytochrome c immunohistochemistry, the TUNEL assay, and M30 immunofluorescence. No statistically significant differences were observed in general histopathological changes, TUNEL, or cytochrome c expression (*p* > 0.050). However, the pigs that received HES 130/0.4 for volume replacement showed significantly higher intensity of the liver M30 immunostaining in the Q-score (*p* < 0.010), H-score (*p* < 0.010), and c indexc index (*p* < 0.050) when compared to animals that received Ringer’s lactate solution or animals in the control group. These findings suggest that HES 130/0.4 induces increased early hepatocellular apoptosis when compared to RL in this model, raising concerns about its hepatic safety profile under haemorrhagic conditions.

## Simple Summary

Hydroxyethyl starch 130/0.4 is a colloid solution used for expanding the plasma volume in situations of severe hypotension that, for example, may result from acute severe blood loss. This volume expansion allows for a more adequate organ perfusion, and consequent oxygen and nutrient delivery to the tissues. Nevertheless, several secondary effects associated with the hydroxyethyl starch 130/0.4 administration have been reported in the scientific literature, mainly when large volumes were used, but its hepatic effect is not clear. In this research, three groups of pigs were used, where just two groups were submitted to acute severe bleeding. In these two groups, volume replacement was either performed with the administration of hydroxyethyl starch 130/0.4 or with Ringer’s lactate. The other group was submitted to the full anaesthetic and monitoring procedures, except for bleeding. After euthanasia, liver samples were collected for histological and immunohistochemical analysis. The liver cells from pigs that had received hydroxyethyl starch 130/0.4 showed significantly early signs of programmed cell death when compared to those that received Ringer’s lactate solution. These findings are of crucial importance when selecting the solution for volume replacement in patients with hepatic disease or that will be submitted to liver transplant.

## 1. Introduction

Several studies have addressed the effects of hydroxyethyl starch (HES) 130/0.4 on kidney histological structure and on renal function, and have been intensively discussed, leading to the suspension of all HES products by the European Medicines Agency (EMA) in 2013 [1]. In addition to the reasons provided by the EMA for this suspension, it is also important to evaluate the HES 130/0.4 effects on other organs besides the kidneys.

The liver is an organ with a unique vascular network. Its blood supply is assured by the arterial branches from the hepatic artery (25% of hepatic blood supply) and by venous branches from the portal vein (75% of hepatic blood supply) in a complex anatomic structure that drains both arterial and venous blood into the sinusoids [2]. Under normal physiological conditions, the pressure within the hepatic arterial and portal venous systems are levelled within the sinusoids. Changes in the portal and hepatic artery blood flow can cause changes in sinusoidal blood flow and pressure, affecting hepatocyte integrity due to the intimate association between sinusoids and the neighbouring hepatocytes [3].

HES 130/0.4 molecules have a hydrodynamic diameter of 15.5 ± 0.7 nm [4], which is significantly smaller than the sieve plate-like pores of the sinusoidal endothelium that allows for exchanging molecules of 150–175 nm between the sinusoidal lumen and the perisinusoidal space (Disse space) [5,6,7], suggesting that HES 130/0.4 molecules may reach the perisinusoidal space and can alter normal hepatocyte health. Healthy non-hepatic capillaries usually allow for the exchange of molecules ranging between 0.5 and 12 nm [5,6].

There are few studies addressing the HES 130/0.4 effects on the liver, and they were performed in ischemia/reperfusion injury [8], in sepsis rat models [9], or in critically ill human patients [10]. Furthermore, to the best of our knowledge, there are no studies addressing the effect of HES 130/0.4 on liver cells in healthy subjects after suffering acute severe blood loss.

We hypothesise that HES 130/0.4 volume replacement in healthy pigs submitted to acute bleeding under general anaesthesia will not cause different histopathologic hepatic effects when compared to pigs in the same conditions that have received Ringer’s lactate (RL) solution.

## 2. Materials and Methods

### 2.1. Animals

The animals were housed separately under controlled conditions of temperature, humidity, air system filtration, and light/dark cycles, and they were fed with a standard diet and tap water ad libitum. All procedures were approved by the Portuguese Ethics Committee for Animal Experimentation (Direção Geral de Alimentação e Veterinária¸ Approval no. 000228). The pigs used in this study were also part of other previously published studies by the same authors [11,12,13], but addressing different objectives.

For this study, eighteen Large White pigs, age three months, were randomly selected for postmortem liver analysis.

### 2.2. Experimental Protocol

Three experimental groups were used: group 1 (RL, *n* = 6), group 2 (HES 130/0.4, *n* = 6), and group 3 (control, *n* = 6). Data comparing all groups included in the present study are reported in the Results section. The experimental procedures were previously described in detail in the studies published by the same research group [12,13,14].

Briefly, pigs were fasted overnight prior the study, but they were allowed free access to water. The animals were premedicated with intramuscular azaperone (4 mg/kg; Stresnil^®^, Janssen Animal Health, Beerse, Belgium) 30 min before the anaesthesia induction. After this, a 22 Ga catheter was inserted in the right auricular vein for drug and fluid administration. Anaesthesia was induced with propofol (4 mg/kg; Propofol 1%, Fresenius Kabi, Bad Homburg, Germany), followed by tracheal intubation and mechanical ventilation. A propofol constant infusion rate of 15 mg/kg per hour was initiated immediately after intubation and remained unchanged during the study. Simultaneously, a 0.3 µg/kg/min remifentanil (Ultiva, GSK, Midlessex, UK) constant infusion rate was initiated and maintained unchanged until invasive monitoring procedures ceased, followed by a 0.2 µg/kg/min constant infusion rate until the end of the study. After reaching stable anaesthesia, a 7 Fr Swan–Ganz optic catheter (Edwards, LifeSciences, Irvine, CA, USA) was introduced in the external jugular vein and progressed into the cranial vena cava. The right femoral artery was cannulated with a 16 Ga catheter (Abbott Animal Health, Abbott Park, IL, USA) for arterial blood removal during the passive bleeding phase of the study. Peripheral oxygen saturation, heart rate, body temperature, and invasive mean arterial blood pressure were monitored using a Datex-Ohmeda S/5 hemodynamic monitor (Datex-Ohmeda, Helsinki, Finland). The RugLoop II Waves software (Demed Engineering, Temse, Belgium) was used to record data every 5 s. After completing all monitoring procedures, pigs from groups 1 and 2 were subjected to a severe acute haemorrhage by passive removal of 30 mL/kg of blood from the right femoral artery over a controlled period of 20 min. The bleeding was followed by a 20 min waiting period, after which a volume reposition period started with 25 mL/kg RL (Lactato Ringer Braun, B. Braun Medical SA, Barcelona, Spain) (group 1) or 20 mL/kg HES 130/0.4 (Voluven, Fresenius Kabi, Bad Homburg, Germany) (group 2). These volumes were administered through the central catheter placed in the cranial vena cava at a constant rate of infusion of 999 mL/h in both groups. After ending the fluid reposition, the animals in groups 1 and 2 remained anesthetised for an additional period of 60 min. The animals in group 3 were submitted to exactly the same anaesthetic and monitoring procedures as animals in groups 1 and 2, except for bleeding and volume replacement. To face naturally occurring fluid losses during anaesthetic procedures, an RL constant infusion rate of 6 + 1 mL/kg/h for each kg above 20 kg of weight [14] was administered to all animals from all groups during all study periods.

### 2.3. Sample Collection and Sacrifice

Following experimental procedures, euthanasia was performed by an intravenous administration of potassium chloride (4 mmol/mL) in the cranial vena cava of all animals. The liver samples were collected postmortem, and a 0.9% sodium chloride solution was used to rinse the tissue samples before immersing them in a 10% neutral-buffered formalin for a maximum period of 48 h. After this time, the tissue samples were prepared for histopathological analysis.

### 2.4. Histological Analysis

Histological analysis was performed in all animals from each experimental group. After fixation, the liver samples were dehydrated through graded ethanol series, embedded in paraffin wax, and 3 µm thick sections were stained with haematoxylin and eosin (H&E). Liver histological evaluation (inflammation, hepatocellular, and vascular changes) was performed by two study-blinded pathologists using light microscopy (Zeiss Axioplan 2 Microscope, Jena, Germany).

### 2.5. Apoptosis Analysis

Apoptotic positive nuclei in liver samples were detected by the M30 Cytodeath immunofluorescence assay, using a mouse monoclonal antibody (M30 CytoDeath Fluorescein, Roche, Mannheim, Germany) for the detection of a caspase cleavage product of cytokeratin 18, and the TUNEL technique, using the in situ apoptosis detection kit (In Situ Cell Death Detection Kit, POD, Roche, Mannheim, Germany), according to the manufacturer’s instructions.

Cytochrome c immunohistochemical detection was performed with the polyclonal antibody cytochrome c (C-20) sc-8385 (Santa Cruz Biotechnology, Dallas, TX, USA) to detect mitochondrial membrane changes consistent with early apoptotic processes. In short, sections were incubated overnight with cytochrome c antibody at 1:3000 dilutions, and then sequentially incubated at room temperature with the labelled (strept)avidin–biotin–peroxidase method (LAB-SA) (ImmunoCruz™ goat LSAB Staining System: sc-2053, Santa Cruz Biotechnology, Dallas, TX, USA). DAB was used for visualisation, followed by haematoxylin counterstaining.

The H-score, the total number of apoptotic cells, the apoptotic index, and the apoptotic cells/mm^2^ were addressed [1,15,16].

### 2.6. Data Analysis

For histological analysis, three different liver zones were observed for grading: zone 1 (which surrounds the periportal region and is richest in oxygen and nutrients; it is less sensitive to oxidant injury by reperfusion), zone 2 (midzonal, and intermediate in oxygen and nutrients), and zone 3 (centrilobular region, where oxygen concentration is low; more sensitive to hypoxia when liver blood supply is compromised). A grade from 0 to 3 was attributed for the liver lesions: grade 0 (absence of lesion), grade 1 (focal or discrete lesions), grade 2 (moderate lesions), and grade 3 (intense lesions) [17,18].

TUNEL, M30 immunostaining, and cytosolic cytochrome c immunolabelling were evaluated using the Quickscore (Q-score) method, which grades staining intensity as follows: undetectable (0), weak (1), moderate (2), intense (3), and very intense (4) [19]. The H-score was calculated for each animal by observing the number of cells with nuclei/apoptotic bodies and its staining intensity in 10 randomly chosen high-power fields observed at 400× magnification.

The H-score ranged from 0 to 400: 0 (absence of reaction), 0–100 (low grade), 100–200 (moderate grade), 200–300 (high grade), 300–400 (very high grade), and >400 (maximum intensity in all cells). Also, for TUNEL and M30 immunostaining, the total number of apoptotic cells in 10 fields was counted and the apoptotic index was calculated (total number of positive cells/total number of cells), and the number of apoptotic cells in each mm^2^ of tissue was also determined [1,15].

The Shapiro–Wilk normality test and Levene’s test were used, respectively, to screen data for normal distribution and for homogeneity of variance. If these assumptions were verified, the data between groups were compared using one-way analysis of variance (ANOVA) and the Bonferroni post-test. Non-normally distributed data were analysed using the Kruskal–Wallis test. Pairwise comparisons were performed using Dunn’s test with Bonferroni correction; the reported *p*-values are adjusted *p*-values.

After data screening for normal distribution and variance, the histology data, the cytochrome C data, and the TUNEL and M30 Q-scores were analysed using the Kruskal–Wallis test, with Dunn’s post-test for multiple comparisons if differences between groups were identified. The remaining TUNEL and M30 data were analysed using one-way ANOVA and Bonferroni post-test for pairwise comparisons. The physiological variables and the duration of the study periods were also analysed using one-way ANOVA. Continuous data are expressed as a percentage or mean ± standard deviation (SD). The *p*-values lower than 0.05 were considered statistically significant.

SPSS for Windows (version 23.0, IBM Corp., Armonk, NY, USA) was used for data comparison.

## 3. Results

### 3.1. General Data

Each group had four males and one female. The animals weighed 27 ± 3 kg in group 1, 27 ± 4 kg in group 2, and 26 ± 5 kg in group 3. No statistical differences were observed between groups in these parameters (*p* > 0.050). The duration of the study periods for all groups are shown in Table 1.

During all study periods, in group 1, the body temperature was 37 ± 1.1 °C (min: 36.2 ± 1.1 °C; max: 37.3 ± 1.1 °C); in group 2, it was 37 ± 1.5 °C (min: 36.3 ± 1.6 °C; max: 37 ± 1.7 °C); in group 3, it was 37 ± 0.6 °C (min: 36.4 ± 0.9 °C; max: 36.6 ± 0.8 °C). The temperature between groups showed a statistically significant difference during all study periods (*p* < 0.001).

In group 1, the heart rate was 78 ± 20 bpm (min: 63.7 ± 15.1 bpm; max: 94.5 ± 27.5 bpm); in group 2, it was 84 ± 23 bpm (min: 68.1 ± 17.12 bpm; max: 108 ± 30.9 bpm); in group 3, it was 80 ± 9 bpm (min: 59.2 ± 17.4 bpm; max: 108 ± 33.2 bpm). The heart rate between groups showed a statistically significant difference during all study periods (*p* < 0.001).

In group 1, the peripheral oxygen saturation (SPO_2_) was 99 ± 2% (min: 94.8 ± 3.8%; max: 99.9 ± 0.3%); in group 2, it was 98 ± 2.4% (min: 94.6 ± 4.5%; max: 99.8 ± 0.5%); in group 3, it was 99 ± 1.7% (min: 98.6 ± 1.3%; max: 99.6 ± 0.7%). There was no statistically significant difference between group 1 and group 2 (*p* > 0.050), but when comparing to the control group, as expected due to the lack of bleeding in group 3, a statistically significant different was observed (*p* < 0.001).

**Figure 1:**
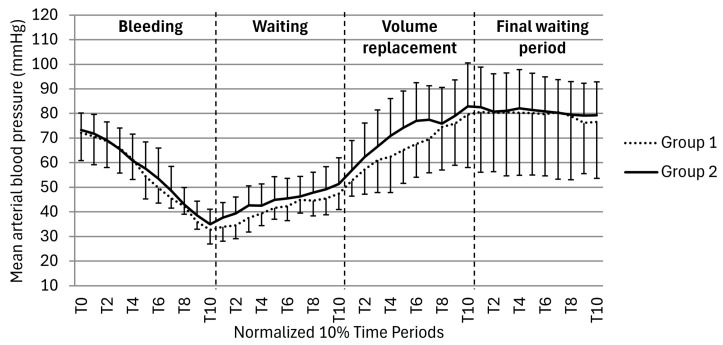
Mean arterial blood pressure during the study periods for groups 1 and 2. No statistically significant differences were observed between groups 1 and 2 (*p* > 0.050).

The trend in mean arterial blood pressure values from animals in groups 1 and 2 during the bleeding, waiting period, volume replacement, and final waiting period of the study are presented in Figure 1. The mean arterial blood pressure in animals from group 3 (control group) was 71.8 ± 5.72 mmHg during all study periods. There was no statistically significant difference between groups during all study periods (*p* > 0.050). When comparing to group 1 and group 2, no statistically significant differences were observed between groups (*p* < 0.050) (Figure 1).

### 3.2. Histological Analysis

**Figure 2:**
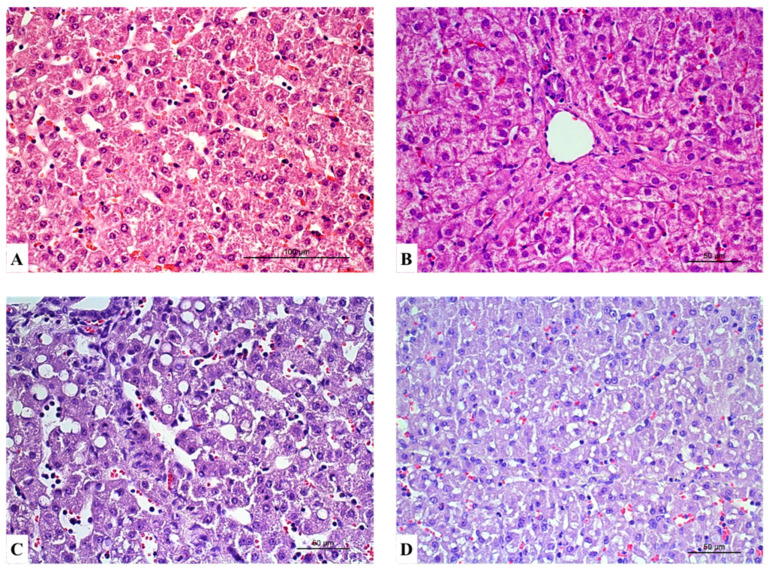
Liver sections showing hepatocellular lesions: hydropic degeneration at grade 1 in the control group 3 (**A**) and grade 2 in the RL group 1 (**B**). Hepatocellular vacuolation at grade 1 observed in the control group (**C**) and grade 2 in the RL group (**D**). Haematoxylin and eosin stain (**A**–**D**). Scale bar = 100 µm (**A**). Scale bar = 50 µm (**B**–**D**).

**Figure 3:**
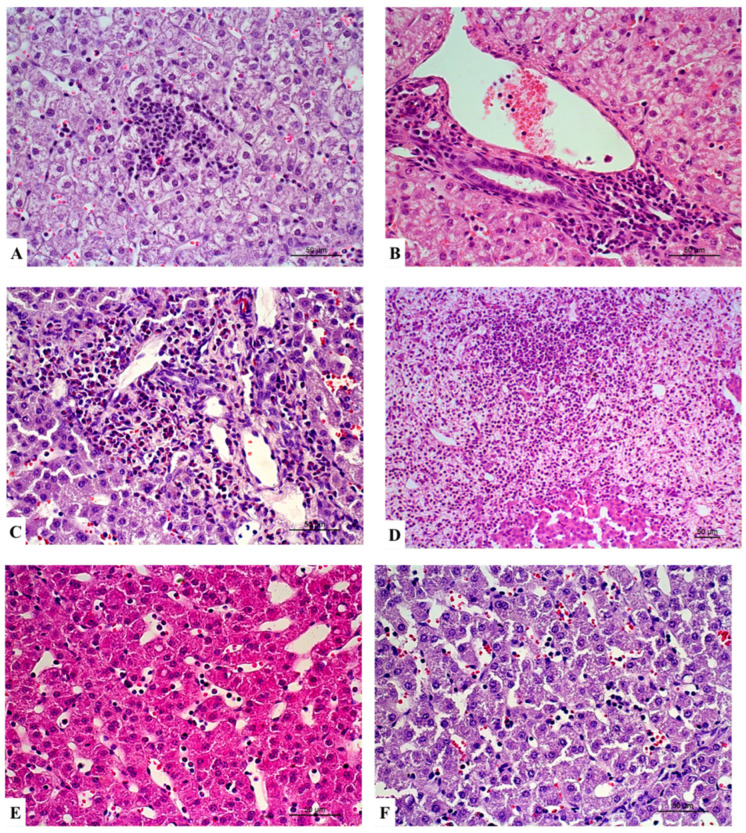
Representative sections of liver with inflammation. Lobular inflammation grade 1 in (**A**) and portal inflammation grade 1 (**B**) in group 1 (RL). Portal inflammation grade 2 in group 3 (control) (**C**) and portal inflammation grade 3 in group 2 (HES 130/0.4) (**D**). Midzonal inflammation grade 1 in group 2 (HES 130/0.4) (**E**) and midzonal inflammation grade 2 in group 3 (control) (**F**). Haematoxylin and eosin stain (**A**–**F**). Scale bar = 50 µm.

**Figure 4:**
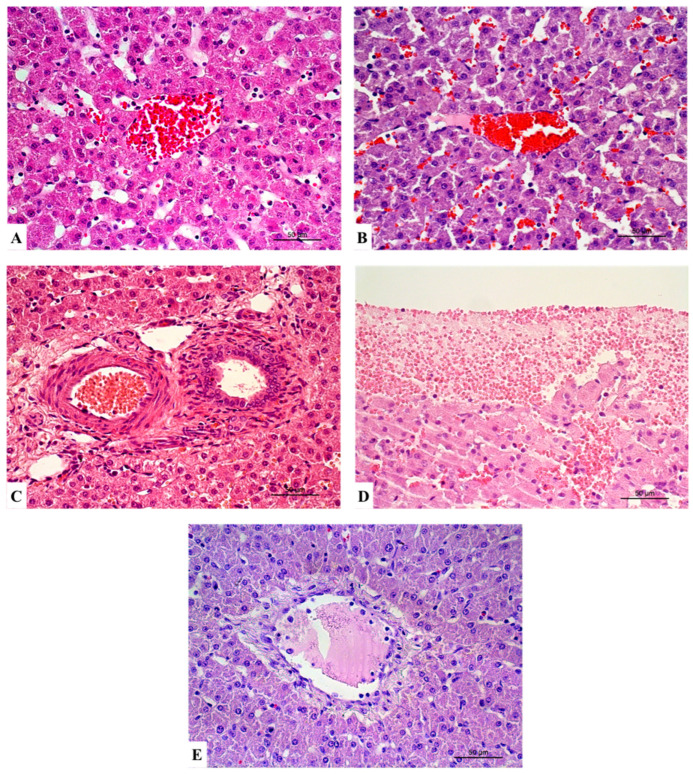
Representative sections of liver with vascular changes. Congestion grade 1 in group 2 (HES) (**A**) and congestion grade 2 in group 3 (control) (**B**). Hyperaemia grade 1 in group 3 (control) (**C**). Haemorrhage grade 1 in group 2 (HES 130/0.4) (**D**). Oedema grade 1 in group 1 (RL) (**E**). Haematoxylin and eosin stain (**A**–**E**). Scale bar = 50 µm.

The histological analysis was performed in the livers of all six animals from each experimental group. Globally, histopathological lesions observed in the liver were subtle. Lesions were categorised as hepatocellular lesions, inflammation, and vascular lesions (Table 2; Figure 2, Figure 3 and Figure 4).

All groups presented animals with centrilobular, portal, and midzonal inflammation. Centrilobular inflammation was of grade 1 in all groups. Animals from group 2 (HES 130/0.4) exhibited sinusoidal inflammation exclusively of grade 1, while animals from groups 1 (RL) and 3 (control) showed sinusoidal inflammation of grades 1 and 2.

Regarding vascular changes, congestion of grades 1 and 2 was observed in all groups.

No statistically significant differences were observed among groups (*p* > 0.050) (Table 2).

### 3.3. Analysis of Pre-Apoptosis and Apoptosis

Pre-apoptotic events were assessed through examination of the immunoexpression of cytochrome c. Apoptosis was assessed through examination of the immunoexpression of M30 and by performing the TUNEL assay. These analyses were performed in all six animals from each experimental group.

**Figure 5:**
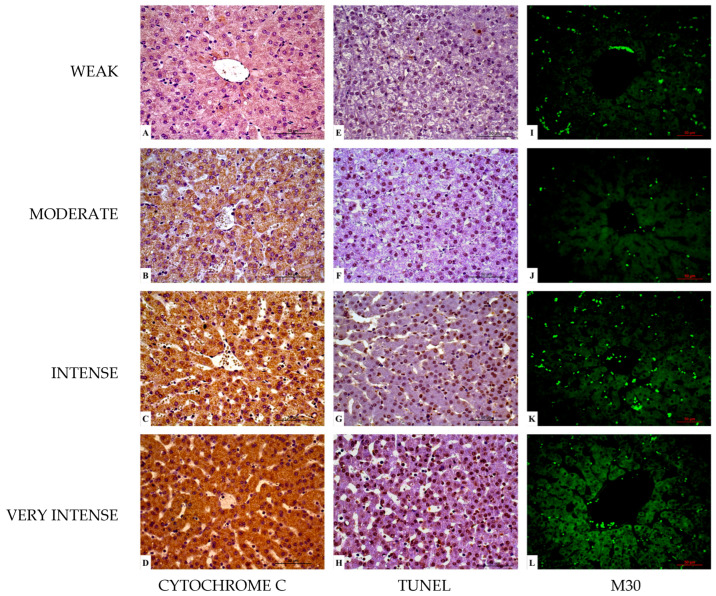
Results for cytochrome c (**A**–**D**), terminal deoxynucleotidyl transferase dUTP nick end labelling (TUNEL) (**E**–**H**), and M30 (**I**–**L**). Weak immunolabelling (Q-score 1) (**A**,**E**,**I**). Moderate immunolabelling (Q-score 2) (**B**,**F**,**J**). Intense immunolabelling (Q-score 3) (**C**,**G**,**K**). Very intense immunolabelling (Q-score 4) (**D**,**H**,**L**). Group 1 (RL; **A**,**B**,**E**,**K**). Group 2 (HES 130/0.4; **F**,**G**,**I**,**L**). Group 3 (control; **C**,**D**,**H**,**J**). Scale bar = 50 µm.

*Cytochrome c:* All animals, independently of the group, showed positive hepatocellular immunostaining for cytochrome c. A cytoplasmatic labelling in hepatocytes, more pronounced in the centrilobular region and less pronounced in the periportal region, was observed in all groups (Table 3; Figure 5A–D). No statistically significant differences were observed among groups (*p* > 0.050).

*TUNEL:* In all animals, the reaction was mainly located in the nucleus, with a higher intensity in the centrilobular region and less intensity in the periportal region (Table 4; Figure 5E–H). No statistically significant differences were observed between groups (*p* > 0.050).

**Figure 6:**
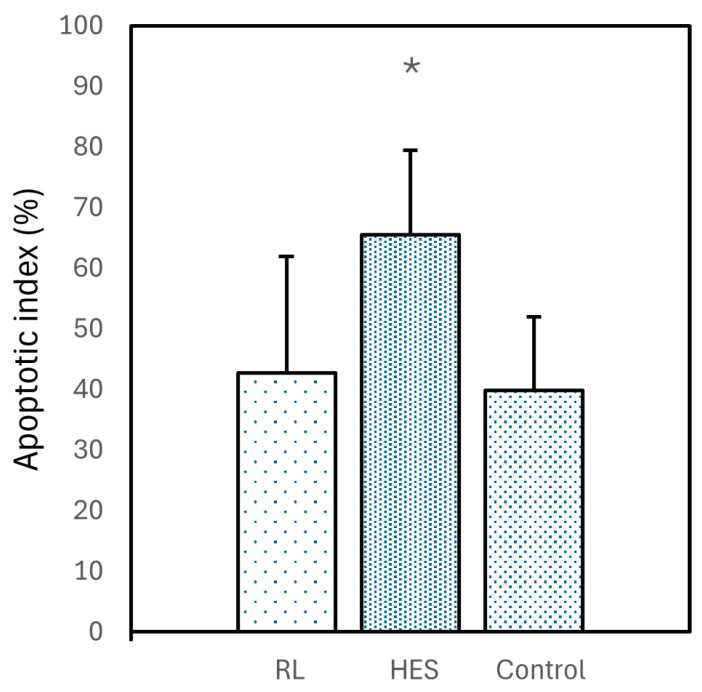
Graphic illustration of the apoptotic index (%) in the livers of the animals in group 1 (RL), group 2 (HES), and group 3 (control). * *p* < 0.050.

*M30:* The reaction was mainly cytoplasmic in hepatocytes, with a higher intensity in the centrilobular region and less intensity in the periportal region. Group 2 (HES 130/0.4) showed a higher intensity in the immunostaining, with statistically significant differences when compared to group 1 (RL) and group 3 (control) (*p* < 0.050) (Table 5; Figure 5I–L and Figure 6).

## 4. Discussion

The null hypothesis was rejected because the animals that received HES 130/0.4 for volume replacement after acute severe blood loss showed a significantly higher intensity of hepatocellular M30 immunostaining for the Q-score (*p* < 0.010), H-score (*p* < 0.050), and apoptotic index (*p* < 0.050) when compared to the animals that received RL or to the animals in the control group.

Previous studies reported that the administration of HES solutions was associated with microcirculatory improvement [20,21,22,23], improved oxygen delivery, decreased peripheral resistance, and improved volumetric flow in the liver sinusoids [9], which may contribute to the reported possible anti-inflammatory effect of HES in different organs and species [20,21,22,23]. Propofol is also known to have anti-inflammatory effects [19]. In our study, the animals in all groups presented different degrees of centrilobular, portal, and midzonal inflammation. As our study was performed in healthy animals, this observed inflammation could have been attributed to systemic inflammation associated to the severe bleeding in animals receiving HES 130/0.4 or RL [24]. However, pigs in the control group were not submitted to bleeding and also showed hepatic inflammation, which suggests that the inflammation observed in the animals is our study could have been due to a pre-existing hepatic light inflammation. Nevertheless, it cannot be completely excluded that the inflammation observed in the livers in all groups could also be related with the anaesthetic procedures. But given the morphology of the inflammatory cells observed (predominantly mononucleated), this inflammation is considered to most likely represent a pre-study background change.

A relation between the presence of underlying inflammation and early apoptosis was considered; however, TUNEL reaction and M30 immunolabelling were observed in numerous regions where inflammation was not visible, and the predominance of reaction and immunolabelling in the centrilobular region, often in the absence of inflammatory cells, does not support inflammation-related apoptosis.

The pre-apoptotic marker cytochrome c, along with apoptotic indicators, M30 immunostaining and the TUNEL assay, were assessed across all experimental groups. Cytochrome c plays a crucial role in mitochondrial electron transport and intrinsic (type II) apoptosis. Its release into the cytosol, where it associates with apoptotic protease-activating factor 1, is a pivotal event in the apoptosis pathway [25,26]. The TUNEL assay detects DNA strand breaks by labelling them through terminal deoxynucleotidyl transferase (TdT) activity [27]. The DNA fragmentation by endonucleases occurs in a late stage in the apoptotic events and for a short time period [28], which indicates that the TUNEL assay does not have a high enough sensitivity to identify changes in early apoptosis. Early in the apoptotic cascade, the occurrence of the cleavage of cytokeratin 18 leads to the formation of a neo-epitope that can be recognised by the M30 antibody [29]. In our study, the animals in group 2 (HES 130/0.4) showed a significantly marked increase in M30-positive immunolabelling when compared to animals that received RL or in the control group, which indicates that HES 130/0.4 may have induced significant hepatocyte early apoptotic changes.

The number of sinusoids in the periportal and centrilobular regions are the same; however, it was verified in the rat liver that the sinusoids are wider and straighter in the centrilobular area when compared to the periportal area, where they are narrower and more tortuous [30]. Thus, blood flow velocity is expected to be smaller in the sinusoidal centrilobular region when compared to the periportal region [4]. The sinusoidal endothelium has sieve plate-like pores that allows for the exchange of molecules between the sinusoidal lumen and the perisinusoidal space [5,6,7]. The width of these pores varies and is smaller in the sinusoidal centrilobular region when compared to the periportal region. However, the percentage of sinusoidal endothelial pores is higher in the centrilobular region [30]. The lower sinusoidal blood flow velocity [3] and the higher open percentage of endothelial fenestra in the sinusoidal centrilobular region may favour a higher exchange of HES 130/0.4 molecules from the sinusoid lumen to the perisinusoidal area in the centrilobular area [30], as the HES 130/0.4 hydrodynamic diameter of 15.5 ± 0.7 nm [4] is considerably smaller than the sinusoidal endothelial fenestra width (150–175 nm) [5,6,7].

In our study, the immunoreaction to the M30 apoptosis marker was more intense in the centrilobular region, and the number of apoptotic cells marked were significantly higher in the animals in group 2, suggesting that these lesions could have been related with HES 130/0.4 accumulation in the perisinusoidal centrilobular area. This possibility is in accordance with the data published by Bagshaw and Chawla [10], who reported an increase in new hepatic organ failure in critically ill patients that received HES when compared to patients that received saline.

Nonetheless, the liver’s response to HES appears to be variable. During cardiopulmonary bypass, HES 130/0.4 limited increases in AST and LDH, indicating hepatoprotection compared to crystalloid priming [31]. However, in sepsis, HES balanced induced substantial hepatic cytokine expression (e.g., a 512-fold increase in ICAM-1), highlighting a potential for hepatotoxicity in inflammatory states [32]. Overall, HES 130/0.4 appears to exhibit a dual profile when considering its inflammatory and systemic effects, dependent on the underlying pathophysiological context.

The nephrotoxic potential of HES 130/0.4 is supported by multiple studies [33,34,35]. A previous study from by Ferreira et al. using a pig model of controlled haemorrhage reported that, even in the absence of elevated BUN or serum creatinine, pigs that were part of a model of controlled haemorrhage receiving HES exhibited significantly greater renal tubular apoptosis and changes indicative of early apoptosis, as indicated by the increased M30 immunoreactivity and cytochrome c expression, respectively [36]. While Schimmer et al. found no significant differences in creatinine or α-microglobulin between groups, molecular markers of renal inflammation (MCP-1 and ICAM-1) were markedly elevated in HES-balance treated animals [32]. Together, these data suggest that HES 130/0.4 may induce subclinical renal injury, emphasising the relevance of tissue-level evaluation beyond standard biochemical markers.

A randomised controlled study by Ortiz et al., using a pig model of controlled haemorrhage, found that fluid resuscitation with HES 130/0.4 resulted in significantly lower percentages of mucosal loss (%ML) in the duodenum, jejunum, ileum, and entire small intestine compared to Ringer’s lactate, indicating that, in the setting of hypovolemia, HES 130/0.4 may offer superior mucosal protection over crystalloids, possibly due to the better restoration of perfusion and capillary oncotic pressure [11].

This study has some limitations. The heart rate, the temperature, and the SPO_2_ were statistically significantly different among the three groups (*p* < 0.001) for the duration of all study periods. These differences are understandable because animals in group 1 and group 2 were submitted to severe acute blood loss, causing a sudden decrease in blood pressure, that directly influenced the heart rate reflex response and body temperature, variables also influenced by the large volume of fluids administered for the volume replacement. But the clinical relevance of this statistical difference is negligible because the minimum and maximum values registered for the heart rate, temperature, and SPO_2_ are within normal physiological thresholds in all groups. Additionally, SPO_2_ and mean arterial blood pressure did not statistically differ between group 1 and group 2 (*p* > 0.050), which assured us that tissue perfusion and oxygen delivery were similar between the RL and HES groups.

The duration of the volume reposition period (minutes) was longer in the group of animals from group 1 when compared to group 2 (*p* < 0.050). This difference results from the different volumes administered to animals in group 1 (RL solution at 25 mL/kg) when compared to group 2 (HES 130/0.4 at 20 mL/kg) at the same 999 mL/h infusion rate.

Also, the study was performed on pigs submitted to acute severe bleeding, followed by a 20 min waiting period before starting the volume replacement. Thus, the hepatic lesions observed were due to transient liver hypoxia followed by volume replacement with RL or HES. This short waiting period may not have allowed the occurrence of severe cellular damage that would lead to mitochondrial lesions, and to a consequent increase in cytochrome c concentrations in the cytosol, and later to DNA fragmentation. This may have also contributed to the non-statistically significant differences between groups observed in our study when using the cytochrome c and TUNEL methods, respectively. Additionally, as the study was performed under controlled experimental procedures, the large standard deviation obtained within groups was unexpected, but it may be considered as the reflex of the interindividual response variability. The large standard deviation and the small number of animals per group facing this standard deviation may have also contributed to not achieving other statistically significant differences between groups in the liver samples observed. Nevertheless, even with a short waiting period, the M30 immunostaining method identified significantly different early hepatic apoptosis in pigs that received HES 130/0.4 when compared to those in group 1 and group 3.

Future research should prioritise direct comparisons between balanced and saline-based HES solutions, incorporate long-term outcomes, and extend the evaluation to clinical populations stratified by disease state and risk for organ dysfunction.

## 5. Conclusions

The administration of HES 130/0.4 for volume replacement in pigs submitted to acute severe bleeding resulted in a significant increase in hepatocellular apoptosis, identified by M30 antibody immunoreactivity, which is expressed in early apoptosis, when compared to pigs that received RL solution or that did not receive any volume replacement. Nevertheless, the data should be critically interpreted due to the study limitations discussed above, mainly the small sample size.

## Figures and Tables

**Table 1:** Duration of the different study periods (minutes) between the end of monitoring procedures and euthanasia in groups 1–3 (data are mean ± SD).

	Group 1	Group 2	Group 3
Duration of bleeding period (minutes) (*p* > 0.050)	20 ± 0.6	21 ± 0.9	--
Duration of waiting period (minutes) (*p* > 0.050)	26 ± 2.3	25 ± 2.9	--
Duration of the volume reposition period (minutes) (*p* < 0.050)	43 ± 5.9 *	35 ± 5.1 *	--
Duration of the final waiting period (minutes (*p* > 0.050)	65 ± 2.7	64 ± 5.9	--
Duration from intubation until euthanasia (hours) (*p* > 0.050)	4 ± 0.5	5 ± 0.6	4.2 ± 0.3

There was a statistically significant difference between groups 1 and 2 in the volume reposition period. * *p* < 0.050.

**Table 2:** Histopathological evaluation of liver damage in all experimental groups (number and percentage of animals affected in each experimental group).

Liver Damage		Experimental Group		
		Group 1 (RL) *n* = 6	Group 2 (HES 130/0.4) *n* = 6	Group 3 (Control) *n* = 6
*Hepatocellular lesions*				
	Hydropic degeneration (*p* > 0.050)	3 (50.0)	1 (16.7)	1 (16.7)
	Hepatocellular vacuolation (*p* > 0.050)	3 (50.0)	0 (0.0)	1 (16.7)
Inflammation				
	Lobular (*p* > 0.050)	2 (33.3)	3 (50.0)	3 (50.0)
	Portal (*p* > 0.050)	3 (50.0)	4 (66.7)	4 (66.7)
	Midzonal (*p* > 0.050)	3 (50.0)	4 (66.7)	4 (66.7)
Vascular lesions				
	Congestion (*p* > 0.050)	5 (83.3)	5 (83.3)	4 (66.7)
	Hyperaemia (*p* > 0.050)	0 (0.0)	3 (50.0)	2 (33.3)
	Haemorrhage (*p* > 0.050)	0 (0.0)	2 (33.3)	0 (0.0)
	Oedema (*p* > 0.050)	1 (16.7)	0 (0.0)	1 (16.7%)

Data are presented as *n* (%). RL—Ringer’s lactate; HES—hydroxyethyl starch.

**Table 3:** Q-score for cytochrome c.

Cytochrome c		Experimental Group		
		Group 1 (RL) (*n* = 6)	Group 2 (HES 130/0.4) (*n* = 6)	Group 3 (Control) (*n* = 6)
Q-score (*p* > 0.050)	0 (undetectable)	0 (0.0%)	0 (0.0%)	0 (0.0%)
	1 (weak)	1 (16.7%)	0 (0.0%)	0 (0.0%)
	2 (moderate)	4 (66.7%)	2 (33.3%)	3 (50.0%)
	3 (intense)	1 (16.7%)	3 (50.0%)	1 (16.7%)
	4 (very intense)	0 (0.0%)	1 (16.7%)	2 (33.3%)
*Mean Q-score*		2.0	2.8	2.8

Data are presented as *n* (%). RL—Ringer’s lactate; HES—hydroxyethyl starch.

**Table 4:** Immunoexpression for TUNEL.

TUNEL		Experimental Group		
		Group 1 (RL) (*n* = 6)	Group 2 (HES 130/0.4) (*n* = 6)	Group 3 (Control) (*n* = 6)
Q-score (*p* > 0.050)	0 (undetectable)	1/6 (16.7)	0/6 (0.0)	2/6 (33.3)
	1 (weak)	3/6 (50.0)	1/6 (16.7)	0/6 (0.0)
	2 (moderate)	1/6 (16.7)	3/6 (50.0)	4/6 (66.7)
	3 (intense)	1/6 (16.7)	2/6 (33.3)	0/6 (0.0)
	4 (very intense)	0/6 (0.0)	0/6 (0.0)	0/6 (0.0)
	*Mean Q-score*	1.3	2.2	1.3
H-score (*p* > 0.050)		62.6 ± 84.6	135.08 ± 75.5	50.5 ± 45.8
Total number of apoptotic cells (*p* > 0.050)		606.2 ± 610.1	1390.5 ± 560.9	677.3 ± 570.8
Apoptotic index (%) (*p* > 0.050)		33.8 ± 35.6	68.7 ± 26.6	29.7 ± 25.3
Apoptotic cells/mm^2^ (*p* > 0.050)		1273.5 ± 1281.1	2920.2 ± 1178.2	1423.0 ± 1199.1

Data are presented as *n* (%) or mean ± standard deviation. RL—Ringer’s lactate; HES—hydroxyethyl starch.

**Table 5:** Immunoexpression for M30.

M30		Experimental Group		
		Group 1 (RL) (*n* = 6)	Group 2 (HES 130/0.4) (*n* = 6)	Group 3 (Control) (*n* = 6)
Q-score (*p* < 0.010)	0 (undetectable)	0/6 (0.0)	0/6 (0.0)	0/6 (0.0)
	1 (weak)	5/6 (83.3)	0/6 (0.0)	4/6 (66.7)
	2 (moderate)	0/6 (0.0)	1/6 (16.7)	2/6 (33.3)
	3 (intense)	1/6 (16.7)	5/6 (83.3)	0/6 (0.0)
	4 (very intense)	0/6 (0.0)	0/6 (0.0)	0/6 (0.0)
	*Mean Q-score*	1.3	2.8 ^a,b^	1.3
H-score (*p* < 0.010)		62.0 ± 39.9	128.3 ± 38.8 ^a,b^	56.8 ± 28.9
Total number of apoptotic cells (*p* > 0.050)		942.2 ± 498.4	1533.5 ± 463.7	936.8 ± 386.3
Apoptotic index (%) (*p* < 0.050)		42.7 ± 19.2	65.5 ± 13.9 ^a,b^	39.8 ± 12.2
Apoptotic cells/mm^2^ (*p* > 0.050)		998.2 ± 514.3	1612.5 ± 487.6	940.1 ± 377.7

Data are presented as *n* (%) or mean ± standard deviation. RL—Ringer’s lactate; HES—hydroxyethyl starch. ^a^ Statistically different from group 1 (RL) (*p* < 0.050). ^b^ Statistically different from group 3 (control) (*p* < 0.050).

## Data Availability

Data available upon request.

## Abbreviations

The following abbreviations are used in this manuscript:

ANOVA	Analysis of variance
EMA	European Medicines Agency
H&E	Haematoxylin and eosin
HES	Hydroxyethyl starch
RL	Ringer lactate
